# Genetic and Environmentally Induced Scalation Variation in Bisexual and Parthenogenetic Lizards

**DOI:** 10.3390/biology15110882

**Published:** 2026-06-03

**Authors:** David Tarkhnishvili, Evsey Kosman, Natia Barateli, Giorgi Iankoshvili

**Affiliations:** 1 Caucasus Leibniz Biodiversity Research Center, Ilia State University, 3/5 Cholokashvili Ave., Tbilisi 0162, Georgia; natia.barateli.1@iliauni.edu.ge (N.B.); giorgi.iankoshvili.1@iliauni.edu.ge (G.I.); 2 Institute for Cereal Crops Research, School of Plant Sciences and Food Security, Faculty of Life Sciences, Tel Aviv University, Ramat Aviv, Tel Aviv 6139001, Israel

**Keywords:** parthenogenesis, lizards, microsatellite genotypes, scalation, phenotypic plasticity, phenotypic variability, genetic variability

## Abstract

Lack of recombination in parthenogenetic organisms limits their ability to adapt to changing environments by natural selection. However, some obligatory parthenogens, such as rock lizards of the genus *Darevskia*, could survive for millions of years across multiple Pleistocene glaciations. There are different explanations for this phenomenon. Analysis of phenotypic variation may shed further light on the high adaptability of the parthenogenetic lizards. We compared the genetic and phenotypic variability of 186 individuals of the parthenogenetic *Darevskia dahli* and 54 individuals of its sexually reproducing paternal species, *D. portschinskii*, whose ranges almost coincide in Georgia (the Caucasus). The analysis showed that, despite the higher genetic variability of the individuals and metapopulations of *D. portschinskii*, phenotypic variability (as measured by KW dispersion and the normalized effective number of individuals per metapopulation), based on the nominal traits, was almost equal in the two species. Moreover, phenotypes of the parthenogen correlated with the distances among the localities, and with the annual rainfall level at a locality. The latter species also had more outlier phenotypes. Phenotypic plasticity may be a strategy for adaptation of parthenogenetic rock lizards, to a certain extent, compensating for the lack of genetic diversity.

## Simple Summary

We compared variation in microsatellite genotypes and scalation in temporal and anal areas in parthenogenetic rock lizard *Darevskia dahli* and its sexually reproducing ancestral species, *D. portschinskii*. The aim of the study was to infer whether a higher genetic diversity of sexually breeding species causes their higher phenotypic diversity, related to the parthenogens. The results of the study were only partly consistent with this expectation. Parthenogenetic *D. dahli* showed minute genetic diversity with no association with the distance among the locations, different from the bisexual species. However, its phenotypic (scalation) variations were associated with both geographic distance between the localities and with the climatic conditions as in the bisexual species. This may be related to the developmental instability in the parthenogens, but it also broadens ecological tolerance in spite of the lack of genetic diversity.

## 1. Introduction

A switch between parthenogenetic and bisexual reproduction occurred multiple times in metazoans [1,2]. Both ways of reproduction have important advantages and no less important shortcomings. Parthenogenetic reproduction allows for the production of more offspring using fewer resources; hence, parthenogenetic populations can grow faster [3,4]. Rapid reproduction allows the parthenogens to expand into the new areas and habitats, especially in higher altitudes with conditions suboptimal for their bisexual relatives [5,6,7]. On the other hand, sexual reproduction ensures a higher diversity of genotypes in a population due to the recombination process. In case of a rapid change in the environment, this may increase the chances of a population surviving in new ecological conditions.

If this basic theory is correct, one can suppose that an optimal adaptive strategy includes alternations of bisexual and parthenogenetic reproduction modes: the former helps the population to survive in extreme or rapidly changing environments, whereas the latter ensures rapid population growth and expansion when the conditions are close to species-specific optimum [3,8]. Indeed, the alternation of asexual and sexual reproduction is commonplace in plants and some animals [1,9,10,11,12,13]. However, many higher animal taxa lack this adaptive strategy [14]. Parthenogenesis in vertebrates is relatively rare. True obligate parthenogenesis is described in sixteen genera of lizards and snakes [15]. No cases are known of regular alteration of parthenogenetic and sexual reproduction in these groups.

In most cases, there are occasional parthenogens produced by bisexual parents [16], which either remain sterile or return to bisexual reproduction, hence with no evolutionary or adaptive consequences [17]. Some obligatory parthenogenetic lineages are produced mainly by distant hybridization, causing possible dysfunction of some processes during meiosis [18]. A return to sexual reproduction in obligatory parthenogens is exceptionally rare. When it occurs, it may involve backcrossing and the production of either asexual or sexually reproducing progeny. Such cases have been documented or hypothesized in some fishes [19,20] and lizards [20,21,22,23,24].

Although there is a lack of theoretical evidence of the unavoidable superiority of sexual breeding [25], its prevalence in most vertebrates and other complex unitary animals is an important argument for the higher fitness of sexual breeders. However, some hybrid parthenogens appear to be quite successful, even in a relatively long geological timespan. Caucasian rock lizards (*Darevskia*), a highly diverse group of species from the Caucasus Mountains [26], have seven parthenogenetic “species” with hybrid origin [27,28,29]. The reproduction of these lizards involves modified meiosis and the production of unreduced eggs [30], and therefore cannot be described either as typical apomixis or typical automixis. The time of their origin is estimated as more than 0.5 Mya [31]. This estimated time suggests that the existing parthenogenetic lineages survived several cycles of Pleistocene glaciation, i.e., dramatic climatic changes. Currently, they coexist with their patrilineal ancestors and are shown to be strong competitors that may even force their parental species from some habitats [32]. Probable reasons for their success are high heterozygosity related to the hybrid origin and fast population growth potential, typical for parthenogens in general.

Phenotypic and genotypic diversity of parthenogenetic *Darevskia dahli* and its patrilineal ancestor *D. portschinskii* were recently analyzed in six river valleys where these species coexist in nature [33]. It was shown that the bisexual species has a higher overall diversity, both genotypic (microsatellite allele-based) and phenotypic, than the parthenogen. It was also established that genetic and phenotypic differences between the metapopulations correlate with the geographic distances in the bisexual species but not in the parthenogen. In that research, the aim was to infer the heritability of individual scalation characters, which could, in the future, help with population analysis with limited use or without the use of genetic markers. However, all analyses were conducted on a metapopulation level, and correlations on the individual level were not studied.

The system, including closely related parthenogens and their parental bisexual species, is potentially ideal for exploring another important evolutionary question: the relation between inherited (genetically determined) phenotypic variation and phenotypic variation that is environmentally induced. Despite the different biological nature of these types of variation, they are difficult to separate—due to the presence of phenocopies [34,35] and the lack of straightforward links between gene and character due to the polygenic genetic architecture of most quantitative characters, as well as pleiotropy and epistasis [36]. Some empirical evidence suggests that selection for best-fit genetically determined phenotypes and phenotypic modification due to the direct environmental impact may have the same direction [37,38,39,40]. However, it is not clear whether phenotypic modification complements the existing genotype-based selection, as was supposed by Tarkhnishvili et al. [33], or whether there is any positive or negative association between them. To answer this question, we extended the analysis of the existing data to reveal the impact of genetic isolation and climatic conditions on phenotypic diversity, focusing on the individual-level study.

## 2. Materials and Methods

### 2.1. Studied System

**Figure 1:**
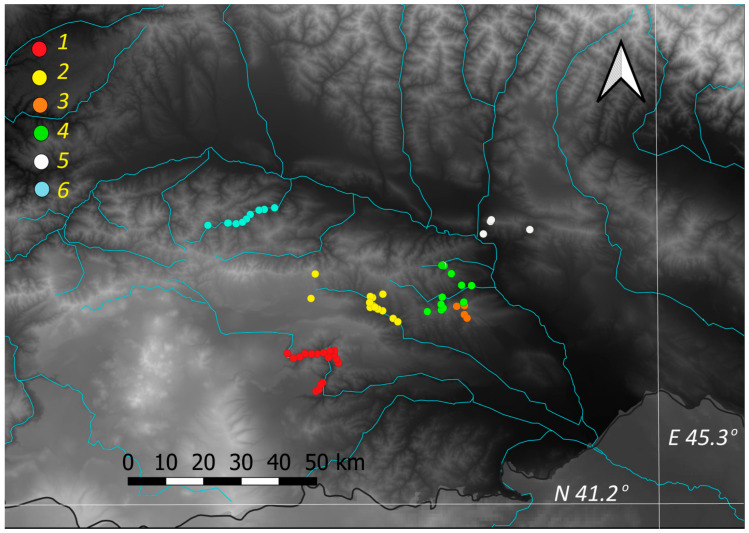
Sampled localities of *Darevskia dahli* and *D. portschinskii.* Individual localities are shown. Metapopulations 1 to 6 are marked with different colors. Rivers shown with blue lines.

**Figure 2:**
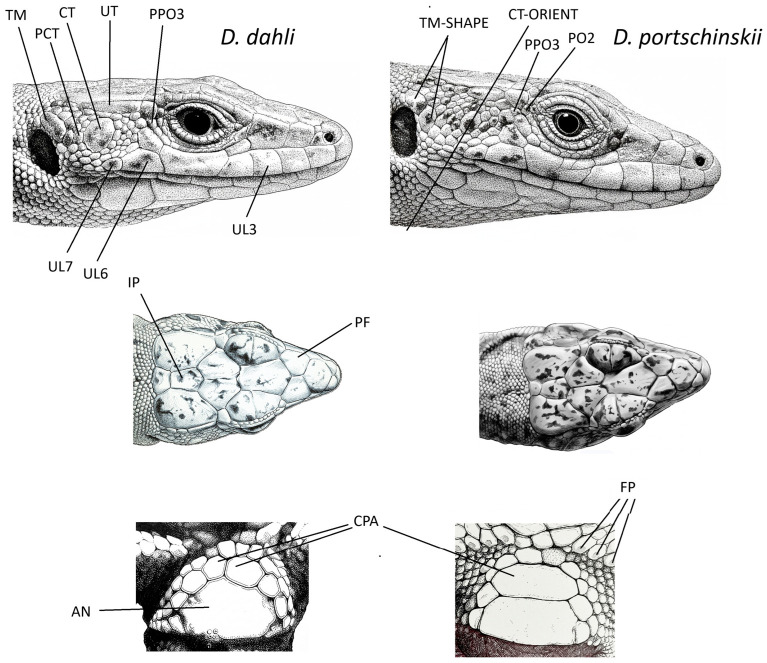
Scales that were used for the phenotypic descriptions. Left panel: *D. dahli*; right panel: female *D. portschinskii*. CT—central temporal; UL—upper labials; UL6—6th upper labial scale; TM—tympanal; UT—upper temporal; UL7—7th upper labial; PPO3—third scale of the second road of post-postorbitals; PCT—post central temporal; PO2—second postorbital; PF—prefrontal; IP—interparietal; AN—anal scale; CPA—preanal; FP—femoral pores.

The research area covered the central part of the Lesser Caucasus east and south of the riv. Mtkvari (Kura) valley and southernmost foothills of the Greater Caucasus in Georgia, where parthenogenetic *D. dahli* and its patrilineal, sexually reproducing ancestor *D. portschinskii* are commonly found in the same locality [32,33]. We sampled *D. dahli* and *D. portschinskii* from 75 localities and six metapopulation areas (Figure 1), corresponding to valleys of smaller rivers, assuming that gene flow within each area is more intensive than between the areas. 186 *D. dahli* and 54 females of *D. portschinskii* were photographed from four perspectives: pileus, right side of the head, dorsum, and anal area (Figure 2). Photographs were taken with a Nikon Coolpix 5400 digital camera (Nikon Corp., Tokyo, Japan) in close-up mode. For small-scale characters, especially on the right side of the head and in the anal area, the camera was positioned approximately 2 cm from the body surface, so that scale boundaries remained sharp and clearly visible. Whenever possible, the photographed surface was oriented perpendicular to the optical axis of the camera to minimize distortion. Individuals were photographed immediately after capture under field conditions.

For part of the caught individuals, tissue samples (tail tips) were collected and stored in ethanol for further DNA analysis. All individuals were released in the same place where they were caught.

Ten nominal and nine numeric characteristics were scored based on the collected images. Microsatellite genotypes were scored from 52 *D. dahli* and 27 females of *D. portschinskii* (Table S1).

### 2.2. Scoring Microsatellite Genotypes

The genotypes of the studied individuals are shown in Table S1. We scored individual lizards at five microsatellite (STR) loci: Du418, Du47, Du218, Du323, and Du215 [41]. All individuals with 2 or 3 missing loci were excluded from further analysis. Microsatellite PCR reactions were performed using the Qiagen Multiplex PCR kit (QIAGEN GmbH, Venlo, Netherlands) with primer concentrations of 0.11–0.14 µM (approximately 0.1 mM). Thermal cycling was performed at 95 °C for 15 min, followed by touchdown of 15 cycles of 94 °C for 30 s, and 56 °C for 45 s, incorporating a stepwise decrease of 0.5 °C at each cycle, 72 °C for 1 min, then 22 cycles of 94 °C for 1 min, 48 °C for 90 s, and 72 °C for 1 min, followed by a final extension at 60 °C for 30 min. PCR multiplexes were developed to reduce the time and cost of genetic analyses. We amplified the five loci in two multiplex PCR reactions using the following primer combinations: Multiplex I—Du47, Du281, and Du418; and Multiplex II—Du323 and Du215. Amplicons were run on a 3130xl Genetic Analyser (Applied Biosystems), using deionized formamide and Genescan size standard LIZ 500 (Applied Biosystems Inc., Foster City, CA, USA). We amplified and scored all loci at least three times and calculated genotyping errors (allelic dropouts, false alleles, and genotype reliability), following the guidelines described in [42,43]. We set the reliable change index (RCI) threshold value as 95%. We verified every individual twice for heterozygosity and three times for homozygosity since allelic dropouts are less likely to be detected at homozygous loci [44].

Microsatellite analysis in this paper was supplementary and inserted for inferring whether there are differences in structure between coexisting populations of *D. dahli* and *D. portschinskii* rather than for inferring the precise population genetic variables; the limited information provided by the five STR loci was sufficient for this purpose.

### 2.3. Describing Scalation

In this study, we use the term scalation to refer to the arrangement, number, and configuration of scales on different body regions; this corresponds broadly to the traditional herpetological term pholidosis.

Thirty-one scalation characters described in ([33]; see the description below) were analyzed from the digital images of 240 lizards from 75 localities. After removing invariant characters and those showing fixed differences between *D. portschinskii* and *D. dahli*, 19 informative traits remained (Figure 2, Table S1); all these characters vary in both taxa. No underlying information on geographic variability was used while selecting the characters.

Nine of the informative traits were numeric (a) the number of scales around the central temporal scale, SC-CT; (b) the number of the upper labials, ULb; (c) the number of the scales between the central temporal and sixth upper labial (CT-UL6); (d) the number of the scales between the tympanal and upper temporal, TM-UT; (e) the number of the scales between tympanal and seventh upper labial, TM-UL7; (f) the number of the scales between the central temporal and the third scale of the second road of postorbitals, CT-PPO3; (g) the number of the scales between the central temporal and the closest upper temporal, CT-UT; (h) the number of the scales in contact with the anal scale, SC-AN; (i) the number of the femoral pores on the left leg (FP). Other ten characters were nominal: (a) the shape of the central temporal scale (round or angular; CT-SHAPE); (b) the orientation of the CT: round, or oval—horizontally or vertically elongated (CT-ORIENT); (c) the structure of the tympanal scale (TM-SHAPE; single or divided); (d) post-CT (PCT) (single or divided); (e) PO2-PPO3—postorbital and post-postorbital (not in contact, in contact, or overlapping); (f) upper labial 3 (UL3; single or divided); (g) CPA-SHAPE—shape of the CPA—absent, small, large, broad; (h) prefrontal (PRF; side smooth, side angled, scale between the prefrontals); (i) interparietal (IP; parallel or angled lines, concaved, broken lines); (j) interparietal thickness (IPT).

### 2.4. Data Analysis

A comparison of genetic and phenotypic characterizations of the same individuals collected from the sampled localities was performed using a reduced sample of 54 *D. dahli* and 27 *D. portschinskii*, for which both phenotypic and genetic data were available. These individuals were also used for the analysis of genetic differentiation among the localities and metapopulations. Individual STR genotypes with missing data at any one locus and phenotypes with up to two missing measurements at each nominal and numeric trait set were included in the data analyses.

We used all studied individuals for estimating the phenotypic distances among the six studied metapopulations: 186 and 54 individual phenotypes of *D. dahli* and *D. portschinskii*, respectively.

The structure and relationships between the lizard metapopulations were analyzed based on a proper measurement of dissimilarity between phenotypes and STR genotypes of individuals. A different mode of reproduction of the two studied species allows for assuming a high level of trait and allele associations for the parthenogenetic *D. dahli* and low or no association for sexually reproducing *D. portschinskii*. The most suitable metrics and approaches for analyzing variability within and among populations of organisms with high levels of trait or allele association are assignment-based methods [45,46,47], whereas average-based methods are more appropriate in cases of no association. Since our objective was to compare phenotypic and genetic variability in populations of *D. portschinskii* and *D. dahli*, which exhibit sexual and asexual reproduction, respectively, both average- and assignment-based approaches were employed in this study.

We considered separately the lizard phenotypes for numeric and nominal traits (9 and 10 traits, respectively). Dissimilarity of the numeric phenotypes was calculated with the mean character difference (*mcd*; pp. 122–123, [48]) after the min-max data standardization of all trait values (for each trait, $v_{stand}=\left(v-v_{min}\right)/\left(v_{max}-v_{min}\right)$, where v, $v_{max}$, and $v_{min}$ are original, maximum and minimum values; ${0\leq v}_{stand}\leq 1$) to guarantee an equal weight of all traits. Differences between the ‘nominal’ phenotypes were measured with the simple mismatch dissimilarity (*m*).

The STR genotypes were compared assuming two models of STR evolution: the Infinite Alleles Model (IAM) and the Stepwise Mutation Model with a variable mutation rate (SMMv). Dissimilarities between the STR genotypes were calculated using the corresponding metrics: *IAM* dissimilarity (Equation (3) in [49]; Equation (6) in [50]) and *SMMv* dissimilarity (Equation (4) in [50]); all mentioned equations were applied for q=2 as for diploids.

#### 2.4.1. Variability Within the Metapopulations

Dispersion of individuals within each metapopulation was estimated using the assignment-based and average-based metrics ${KW=KW}_{\rho }$ and ${ADW=ADW}_{\rho }$, respectively, with regard to the relevant dissimilarity *ρ* between individual profiles [45,46,47], where ρ=mcd, m for the numeric and nominal phenotypes, respectively, and ρ=IAM, SMMv for the IAM and SMMv models of STR evolution.

The effective number of different individuals (*ENDI*) within a metapopulation of size *N* was estimated with the metric of functional trait dispersion (Equations (5) and (6) in [51]): *ENDI* = ^1^*D*(*T*, *M*) for the corresponding dispersions $M={KW=KW}_{\rho }$ and $M={ADW=ADW}_{\rho }$ in the case of assignment- and average-based approaches, respectively; ENDI ranges from 1 to *N*. Since the effective number depends on the actual number of individuals sampled in a given metapopulation, the normalized version of this indicator *nENDI* = ^1^*nD*(*T*, *M*) was considered (corrected Equation (5) in [52]; Equation (3) in [53]). Note, values of nENDI belong to the $\left[0,\;1\right]$ interval; nENDI estimates are independent of a metapopulation size and, therefore, allow for comparison of variability within metapopulations of different sizes.

#### 2.4.2. Uniqueness of Individuals and Metapopulations

Uniqueness of each individual phenotype (numeric and nominal) and STR genotype (for the IAM and SMMv models of STR evolution) was estimated separately for *D. portschinskii* and *D. dahli* with the singularity metric based on the corresponding dissimilarity between the phenotypes/genotypes (Equations (1)–(3) in [54]). Then the singularity of a metapopulation was calculated as the average singularity of all phenotypes that belong to it (Equations (7) and (8) in [54]).

#### 2.4.3. Variability Among the Metapopulations

Distance between the metapopulations was calculated using the assignment-based and average-based metrics ${KB=KB}_{\rho }$ and ${DAD=DAD}_{\rho }$, respectively, with regard to the relevant dissimilarity *ρ* between individual profiles [45,46,47].

Differentiation among the metapopulations was estimated with the permutation test (1000 random partitions) for differentiation statistics ${dif}_{KW}$ and ${dif}_{ADW}$ (Equation (1) in [55,56]) for the assignment- and average-based dispersions KW and ADW, respectively (see also [53]; Equation (13)) and Kosman et al. ([57], p. 565)].

The effective number of different metapopulations (*ENDP*) for the assignment-based (*KB*) and average-based (*DAD*) distances between them and the corresponding estimates of the extent of structural differentiation of metapopulations were obtained according to Equations (7)–(9) and Equation (10) in [58].

#### 2.4.4. Association of Dissimilarities Between Phenotypes, Genotypes and Environmental Conditions

Association of relationships between numeric phenotypes, nominal phenotypes, STR genotypes for IAM and STR genotypes for the SMMv model was analyzed separately for *D. portschinskii* and *D. dahli*. The associations were first explored using the Mantel test with pairwise distance matrices obtained for all compared variables. However, because the Mantel tests may have limited power and may be sensitive to spatial non-independence [59], we additionally applied distance-based redundancy analysis, dbRDA, based on principal coordinates [60]. Phenotypic dissimilarity matrices were analyzed at the locality level separately for each species and for the numeric and nominal traits. Geographic coordinates of sampling localities were used to generate distance-based Moran’s eigenvector maps, dbMEMs, representing spatial structure at different scales [61]. Environmental predictors were the mean temperature of the warmest quarter (BIO10), and annual precipitation (BIO12), scored from WorldClim Version 2.1 (http://www.worldclim.org/ (accessed on 26 May 2026)), which is a set of global climate km^2^ layers (climate grids) with a spatial resolution of 1 [62]. Both variables were standardized before analysis. Candidate dbMEM spatial predictors were subjected to forward selection and retained only if significant. The significance of the global dbRDA models and individual environmental terms was assessed by permutation tests. The dbRDA/dbMEM analyses were used as the principal test of environmental association.

#### 2.4.5. Association Analysis of Individual and Climate Differences, and Comparison of Phenotypic, Genotypic and Geographic Distances Between Metapopulations

A comparison of individual phenotype and genotype dissimilarities on the individuals and on the locality levels with differences in temperature and rainfall in exact sampling localities of those individuals was done using the Mantel test. This test was also applied to analyze the association of the assignment-based (${KB}_{\rho }$) and average-based (${DAD}_{\rho }$) distances between the metapopulations of *D. portschinskii* and *D. dahli* and the distances between localities of those metapopulations (dissimilarity ρ between individuals corresponded to each data type).

### 2.5. Software Used

The MxComp program of the NTSYSpc package (version 2.2; Exeter Software, Setauket, NY, USA) was used for performance of the Mantel test. dbRDA and dbMEM analyses were performed in R (version 4.6.0) using the packages *vegan* [63] and *adespatial* [64]. Dissimilarities between STR profiles were calculated using the LOCUS software (accessed on 26 May 2026). Other above-mentioned calculations were performed with the FUNCTIONAL DIVERSITY ANALYSIS TOOLS (FDAT) software (accessed on 26 May 2026). The LOCUS and FDAT packages are available at https://en-lifesci.tau.ac.il/profile/Kosman (accessed on 26 May 2026). Standard statistical analyses and visualization of data were performed with Excel 16.0 [65] and SPSS v. 29 [66].

## 3. Results

It is very challenging to compare population variability (genetic or functional) of organisms with different modes of reproduction because proper approaches and metrics for the corresponding analyses are not generally the same: the assignment-based methods are suitable for asexually reproducing organisms (e.g., *D. dahli*), whereas in the case of sexual propagation (e.g., *D. portschinskii*), the average-based methods can be used for populations with completely random mating (panmixia) and no association among genes or traits. Since there is no evidence that the studied *D. portschinskii* populations met the latter two conditions, a comparative study of *D. dahli* and *D. portschinskii* with the assignment-based methods seems more reasonable, and, therefore, the corresponding results are reported here. Note, however, that the average-based approaches were also applied in this study and provided rather qualitatively similar outcomes.

### 3.1. Variability Within Each Species and Metapopulations

Genetic variability within *D. portschinskii* was higher than for *D. dahli*: estimates of the *KW* dispersion and the normalized effective number of different individuals (*nENDI*) were 0.349 vs. 0.083 and 0.339 vs. 0.072, respectively, for the *SMMv* dissimilarity between SSR genotypes (Table 1). Phenotypic variability based on numeric traits was also significantly higher in *D. portschinskii,* with the *nENDI* estimate 0.298 vs. 0.188. For the nominal traits, estimates of variability were nearly equal (the corresponding *nENDI* values were 0.841 for *D. portschinskii* vs. 0.836 for *D. dahli*; Table 1). In individual metapopulations, the genotypes and numeric phenotypes within *D. portschinskii* were also much more variable than for *D. dahli*, whereas the direction of the differences between the two species based on nominal characters varied (in three out of six metapopulations, *nENDI* estimates were higher for one of the species; Table 1).

### 3.2. Differentiation Among Species and Metapopulations

Differentiation among the six metapopulations of each species was also significant for all types of data (Table 2). The extent of differentiation (*ED*) among the metapopulations was higher in *D. portschinskii* for the STR genotypes as well as for both the numeric and nominal phenotypes (e.g., *ED* = 0.180 vs. 0.045 for the SSR genotypes assuming the SMMv model, and *ED* = 0.368 vs. 0.282 for the nominal data; Table 2). For each species, differentiation among its metapopulations was more substantial for nominal characters than for the numeric ones.

The relative range of variation, $\left(max-min\right)/max$, of the metapopulation-specific *nENDI* values based on the numeric phenotypes was higher in *D. portschinskii* than in *D. dahli*: 0.230 versus 0.183. For the nominal phenotypes, the situation was reversed: 0.169 in *D. dahli* versus 0.056 for *D. portschinskii* (follows from Table 1). For the SSR genotypes (SMMv model), the relative range of variation in the *nENDI* values was much larger for *D. dahli*: 0.68 versus 0.38.

### 3.3. Uniqueness of Individuals and Metapopulations

**Figure 3:**
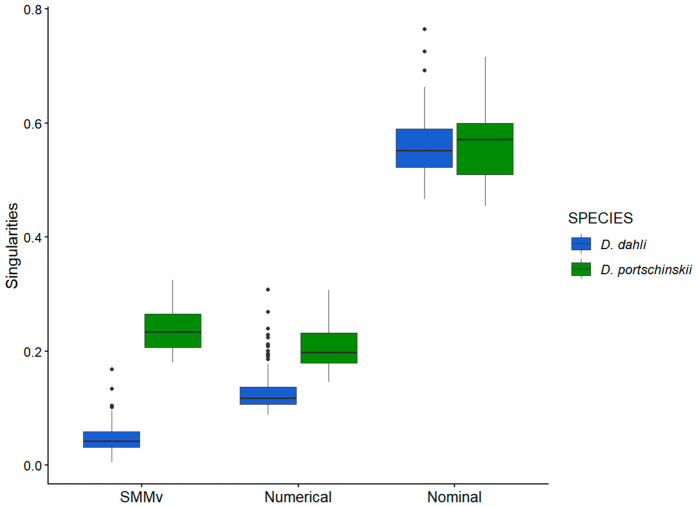
Boxplots showing the distribution of the normalized singularity values for *D. dahli* and *D. portschinskii*. Middle line inside the box—median value; boxes—50% of values; whiskers—1.5 interquartile ranges; o—outliers; ∙—extreme outliers (>3 interquartile range).

Singularity analysis of *D. portschinskii* did not reveal individuals of this species with an extreme genotype or phenotype except for one numeric phenotype: for the SSR genotypes, the spectrum of normalized singularity estimates was ‘continuous’ without significant gaps at the top of the list of individuals, ranging in decreasing order of their singularity (Table S2). Although the average and median singularity measures based on the numeric phenotypes were higher in *D. portschinskii*, the relative variability or relative range of the spectrum of the corresponding singularity values (e.g., in terms of coefficient of variation) was higher for *D. dahli* (Table 3 and Table S2; Figure 3). Singularities based on the nominal traits showed nearly equal values for *D. portschinskii* and *D. dahli* (Table 3).

Both the genetic and numeric phenotypic singularities showed the presence of outliers in *D. dahli* but to a lesser extent in *D. portschinskii* (Figure 3; Table S2). Genotypic outliers in *D. dahli* are explained by identical or nearly identical genotypes in most of the studied individuals; even a few de novo mutations can make an individual unique and highly dissimilar from the others. However, the phenotypic singularity indicates the presence of deviant phenotypes specific to individual local habitats. Note, a large number of *D. dahli* individuals with very low estimates of genotypic and phenotypic (numeric) singularities (bottom of the lists in Table S2) means a high level of genotypic and phenotypic redundancy in the parthenogen.

### 3.4. Association Between Individual Phenotypic and Genotypic Differences

At the individual level, there was no significant association between genetic and phenotypic dissimilarities in either of the two studied species for the *SMMv* dissimilarity estimates between STR genotypes (using the SMMv model of STR evolution) and for two types of phenotypes based on numeric and nominal traits, which is not unexpected considering the limited genetic data and neutrality of STR loci. For both *D. portschinskii* and *D. dahli*, the relationships between individuals based on numeric and nominal phenotypes did not concur (*p* > 0.05).

### 3.5. Association of Genotypes and Phenotypes with Geography and Environmental Variables

A significant association between genetic differences and geography was established in *D. portschinskii*, but not in *D. dahli*. In the former species, genotype *SMMv* dissimilarities of individual lizards correlated significantly with geographic distances between individual localities (the coefficient of Mantel correlation was 0.38, *p* < 0.001). In contrast, in *D. dahli*, the correlation coefficient was close to zero and insignificant.

The outcome of correlations between the phenotypic differences among the individuals versus differences in their exact localities and the environmental variables is shown in Table 4. In *D. dahli*, phenotypic differences calculated from the numeric data weakly, albeit significantly, correlated with the geographic distances among the individuals and with disparities of the average rainfall levels at the corresponding localities. On the level of individuals, the correlation of the geographic distance with phenotypic dissimilarity, based on both nominal and numeric traits, was significant for both species. In *D. portschinskii*, dissimilarities between nominal phenotypes of individuals showed a weakly significant Mantel correlation with differences in temperature of the warmest quarter (BIO10).

The dbRDA results partly supported the Mantel-test patterns (Table 5). Forward selection did not retain any significant dbMEM spatial eigenvectors; therefore, the final dbRDA models included average summer temperature (BIO10) and average annual rainfall (BIO12) as environmental predictors without additional dbMEM covariates. In *D. dahli*, the dbRDA model for numeric phenotypes was significant, but explained only a small proportion of variation: R^2^ = 0.051, adjusted R^2^ = 0.015, *p* = 0.005. Term-level tests indicated that this weak climatic association was mainly related to the average annual rainfall.

In *D. portschinskii*, dbRDA detected a significant association between numeric phenotypic variation and the combined climatic predictors BIO10 and BIO12: R^2^ = 0.185, adjusted R^2^ = 0.125, *p* = 0.001. Thus, the climatic component in *D. portschinskii* was more evident in the multivariate dbRDA framework than for individual climatic variations.

dbRDA models for nominal phenotypes were not significant overall in either species, indicating that the climatic association was mainly restricted to numeric traits.

## 4. Discussion

In this paper, we intended to determine the extent to which genetic isolation and climatic conditions affect the phenotypic diversity of the bisexual *D. portschinskii* and the parthenogenetic *D. dahli*. Despite the limited genetic data (sample size and number of STR loci), some important preliminary results were obtained and reported here. The further discussion clearly demonstrates and justifies the need for a deeper genetic study of coexisting parthenogenetic and bisexual *Darevskia*, aimed at analyzing to what extent genetic interactions and the direct influence of environmental conditions on lizard development shape phenotypic variability.

Not surprisingly, the overall genetic variation was higher in the recombinant population of the bisexual species than in its daughter parthenogen. In the bisexual *D. portschinskii*, but not in the parthenogenetic *D. dahli,* the pairwise genetic distances among metapopulations correlated with the corresponding distances among the studied areas, exhibiting a typical isolation-by-distance pattern. Tarkhnishvili et al. [33] showed that the fixation index (*Rst*) between pairs of *D. portschinskii* metapopulations from eastern Georgia correlates with geography. In the present study, we revisited the hypothesis of isolation by distance, based on genetic distances between metapopulations, and also considered the dissimilarities between STR genotypes of individuals versus the distances between the exact localities of the corresponding individuals. Such segregation is typical of a population structure that has been established for a sufficiently long time [67,68,69]. Considering the significant correlation between geographic distance and genetic dissimilarity among individuals in relatively close localities (a road distance of up to approximately 130 km), we conclude that migration rates between populations are low and decrease rapidly with distance.

Genetic variation in the parthenogenetic *D. dahli* was low. No correlation was found between geographic distances and genetic differences, neither for metapopulations nor for separate individuals. The complete absence of association of this genetic variability pattern of *D. dahli* with geographic separation may result from the recent expansion of this form throughout the studied area from a single center [70]. This explanation aligns with considering parthenogens as opportunistic “weed” species [71,72,73]. Females of *D. dahli* lay more but smaller eggs than *D. portschinskii*, which is also typical for the “weed” reproduction strategy [73].

The overall variation based on the numeric phenotypes was also higher in *D. portschinskii* than in *D. dahli,* reflecting a substantial genetic component of this variation (see also [33]). This trend was observed for the species both for the overall samples and for individual metapopulations; *D. portschinskii* also showed higher differentiation among the metapopulations than its parthenogenetic daughter species. The analysis based on the nominal characters did not show such strong differences, although differences among metapopulations were again significantly higher in *D. portschinskii***.** In contrast, singularity analysis showed that the number of outliers was higher in *D. dahli* for both numeric and nominal phenotypes.

Different from the genetic distances, phenotypic dissimilarities between the individuals correlated with the geographic distances between the individuals’ exact localities in both the sexually reproducing and the parthenogenetic species. However, the biological interpretation of this pattern differs for the two species. In the parthenogenetic *D. dahli*, the absence of a comparable association between genetic and geographic distances suggests that the geography-dependent phenotypic variation is unlikely to represent isolation by distance. Instead, it probably reflects environmental differences among geographically distant localities. This interpretation is supported by the significant association between numeric phenotypic differences in *D. dahli* and differences in average annual rainfall, detected both at the individual and locality levels. The dbRDA analysis also supported this hypothesis to some extent, although the explained variation was small.

In the sexually reproducing *D. portschinskii*, phenotypic geographic structure is more difficult to interpret because genetic distances also increased with geographic distance. Thus, phenotypic differences among distant localities may partly reflect spatial genetic differentiation, environmental effects, or both. The Mantel test provided only weak evidence for simple climatic correlations in this species, whereas the dbRDA analysis indicated an association between numeric phenotypic variation and the combined climatic gradient BIO10–BIO12 (summer temperature—annual rainfall). Therefore, unlike *D. dahli*, the climatic component of phenotypic variation in *D. portschinskii* cannot be clearly separated from the geography-dependent genetic structure. These results suggest that rainfall/humidity-related environmental effects are the most plausible explanation for numeric phenotypic differentiation in clonal *D. dahli*, whereas in sexual *D. portschinskii*, phenotypic variation probably reflects a mixed impact of genetic and environmental spatial structuring.

These results lead us to the conclusion that the environmentally induced variation in *D. portschinskii* is at least not higher than in the parthenogenetic form, and even conversely, phenotypes of *D. dahli* may change more strongly under the effect of some environmental variables, e.g., humidity.

Considering the minute genetic variation in *D. dahli*, the correlation of numeric phenotypes with local climates should result from phenotypic plasticity. Therefore, it appears that the humidity affects an individual developmental pathway (e.g., egg development) in the parthenogen more than in its parental sexually reproducing lizard. The question remains whether this high developmental plasticity may increase the adaptability of *D. dahli* to changing conditions, compensating for the lack of recombinant variation. The adaptive power of a broad norm of reaction is well-known [74,75], although it is not universal. For instance, intensive UV radiation increases the intensity of pigmentation in Cladocerans, but this can potentially harm the population, making individuals more vulnerable to predation by fish [76]. High phenotypic plasticity may reflect an imbalance and weakening of developmental homeostasis [77,78]. In our case, numeric characters are associated with the number of scales in some parts of the temporal and anal areas. At first glance, this should have, if any, a minute impact on the survival of individuals. On the other hand, Sakich and Tattersal [79] showed that reduced scalation in bearded dragons (*Pogona vitticeps*) leads to faster dehydration; this may decrease viability in warmer habitats. Scalation characters may also correlate with more important traits, such as the body size of juveniles. Remarkably, a higher phenotypic plasticity of parthenogens versus their sexually reproducing relatives is not universal, even in lizards. Kearney & Shine [6], who studied the effect of incubation temperature on the phenotype (including scalation) of sexually reproducing and hybrid parthenogenetic geckoes (*Heteronotia*), observed the effect opposite to our results: reduced phenotypic plasticity of the hybrid parthenogenetic lineage.

In any event, a considerable phenotypic plasticity of a parthenogen requires an explanation or, at the very least, a hypothesis underlying this fact. The first, mechanistic explanation is developmental instability caused by additive genetic variation [80,81,82]. All parthenogenetic *Darevskia* originated from hybrids of evolutionarily distant bisexual species [27,33,83]. The estimated divergence time of the two parental species of *D. dahli*, *D. portschinskii* and *D. mixta* exceeds 10 Myr [84,85]. We should expect multiple differential changes in genomic architecture aggregated during the period of divergence between *D. portschinskii* and *D. mixta*. These genome transformations may cause dysfunction in multiple genes [86], including those responsible for the normal meiosis process, as well as other features, such as the control of embryonic development in the hybrid. It has been previously demonstrated that certain developmental malfunctions occur in these forms, including abnormal phenotypes [87] and twinning during egg development [88]. The presence of multiple phenotypic outliers in *D. dahli,* revealed by singularity analysis, is likely a reflection of developmental instability; an alternative explanation is the hypothetical presence of triploid backcrosses, as in some other populations of this parthenogen [89]. In contrast to our results, the low phenotypic variation in hybrid parthenogenetic *Heterodontia* can be explained by a much more recent split among their parental species compared to the parthenogenetic *Darevskia* [90].

An alternative (or, rather, supplementary) explanation is that the phenotypic plasticity, when it does not lead to the development of abnormal phenotypes, may increase the adaptive potential of *D. dahli*. Multiple studies are exploring the phenotypic plasticity of reptiles, which is dependent on incubation temperature [91,92,93]. Most of these studies show little effect of the incubation temperature on morphological traits such as body size or relative limb length. In contrast, the impact of temperature on egg development, growth rates, performance, and survival is substantial [92]. None of the 175 studies summarized in Noble et al. [92] described the phenotypic plasticity of scalation characters. Variation in scalation characters in *D. portschinskii* aligns with this general finding, suggesting that phenotypic plasticity (dependence of the scalation traits on local climate) is closely related to the genetically determined variation expressed in the differences associated with isolation by distance. However, the situation in parthenogenetic *D. dahli* is the opposite, due to high phenotypic plasticity, which is supposedly related to its hybrid origin and additive genetic variation.

The variability of individuals based on the nominal traits was higher in *D. dahli* than in *D. portschinskii*, and the former species had more deviant phenotypes. Since the genetically based variability within and differentiation among metapopulations associated with isolation were higher in the bisexual species, phenotypic plasticity makes a critical contribution to the phenotypic variability of *D. dahli*. A significant association of variation in numeric traits in this species with the temperature of a locality suggests that the phenotypic plasticity of this parthenogenetic form is directed by a temperature significantly stronger than that of *D. portschinskii*.

Pfenning et al. [75] suggested that phenotypic plasticity can significantly contribute to adaptive evolution. The increased fitness can be achieved by canalizing a particular developmental pathway (genetic assimilation or genetic accommodation) and, conversely, by widening the reaction norm. If parthenogens can evolve a broader relative reaction norm, and their phenotypic plasticity leads to the development of the most singular outlier phenotypes, this could compensate for the lack of rapid genotypic variation and, hence, the population’s inability to adapt rapidly to changing environmental conditions through natural selection. In this case, the evolution of broadening reaction norms does not require substantial genetic change and can be driven by simple dominant alleles that can arise during the mutation process.

The mechanism of adaptation through phenotypic plasticity in parthenogens remains unclear and requires detailed genomic studies in the future. However, the survival of the parthenogenetic species throughout multiple glacial cycles [31,83], as well as their ability to outcompete their sexually breeding relatives, suggests that the broadening of reaction norms can be a highly efficient adaptation mechanism.

## 5. Conclusions

The results of our study suggest that scalation patterns in rock lizards may vary in response to local climatic conditions, particularly annual rainfall and average summer temperature. The association with annual rainfall was at least as strong, and possibly stronger, in the parthenogenetic species *Darevskia dahli* than in its sexually reproducing paternal species, *D. portschinskii*. Because *D. dahli* showed no evidence of isolation by distance, this pattern suggests that environmentally induced phenotypic variation may be an important component of its ecological differentiation and adaptation.

The developmental plasticity observed in the parthenogen may result partly from weakened developmental homeostasis. *Darevskia dahli* is of hybrid origin, and its parental lineages separated more than 10 million years ago; such deep divergence may contribute to developmental instability. This interpretation is indirectly supported by the presence of multiple phenotypic outliers in populations of *D. dahli*. At the same time, the potentially negative effects of unstable development may be partly compensated by a stronger developmental response to climatic variation. Such plasticity may help parthenogenetic lineages persist under changing environmental conditions despite their low genetic diversity.

## Figures and Tables

**Table 1:** Variability within the studied metapopulations of *D. dahli* and *D. portschinskii*.

	*D. dahli*					*D. portschinskii* ^a^		
		*KW* Estimate ^b^/*nENDI* Estimate ^c^				*KW Estimate*/*nENDI Estimate*		
Site	N	SMMv ^d^	Numeric ^e^	Nominal ^f^	N	SMMv	Numeric	Nominal
1	13	0.051/**0.037**	0.173/**0.132**	0.771/**0.556**	*2*	*0.228/**0.228***	*0.235/**0.187***	*0.733/**0.613***
2	8	0.091/**0.088**	0.184/**0.132**	0.816/**0.570**	*4*	*0.312/**0.307***	*0.210/**0.202***	*0.800/**0.650***
3	13	0.055/**0.048**	0.164/**0.128**	0.756/**0.555**	*6*	*0.207/**0.203***	*0.284/**0.243***	*0.533/**0.440***
4	6	0.125/**0.115**	0.163/**0.116**	0.787/**0.570**	*4*	*0.220/**0.208***	*0.237/**0.192***	*0.775/**0.577***
5	9	0.054/**0.041**	0.155/**0.138**	0.686/**0.588**	*7*	*0.338/**0.328***	*0.215/**0.193***	*0.643/**0.581***
6	5	0.074/**0.067**	0.168/**0.142**	0.678/**0.557**	*4*	*0.206/**0.201***	*0.260/**0.220***	*0.650/**0.518***
Mean		0.075/**0.066**	0.168/**0.131**	0.749/**0.566**		*0.252/**0.246***	*0.240/**0.206***	*0.689/**0.563***
Pool	54	0.083/**0.072**	0.192/**0.188**	0.834/**0.836**	*27*	*0.349/**0.339***	*0.297/**0.298***	*0.830/**0.841***

^a^ all estimates for *D. portschinskii* are shown in *italics*; ^b^ assignment-based dispersion *KW*; ^c^ normalized Effective Number of Different Individuals (*nENDI*) based on the *KW* dispersion (shown in **bold**); ^d^ results for STR genotypes assuming the Stepwise Mutation Model with variable mutation rate (SMMv) of STR evolution; ^e^ results for numeric phenotypes (numeric traits); ^f^ results for nominal phenotypes (nominal traits).

**Table 2:** Differentiation among the studied metapopulations of *D. dahli* and *D. portschinskii*.

	*D. dahli*/*D. portschinskii* ^a^		
	SMMv ^b^	Numeric ^c^	Nominal ^d^
Coefficient of differentiation	0.020/**0.120**	0.019/**0.063**	0.059/**0.149**
Significance of differentiation (*p*-value)	0.001/**0.010**	0.001/**0.001**	0.001/**0.022**
*ENDP* ^e^	1.22/**1.90**	1.45/**1.99**	2.98/**3.58**
*ED* = *nENDP* ^f^	0.045/**0.180**	0.064/**0.141**	0.282/**0.368**

^a^ estimates for *D. portschinskii* are shown in **bold**; ^b^ results for the STR genotypes assuming the Stepwise Mutation Model with variable mutation rate (SMMv) of STR evolution; ^c^ results for the numeric phenotypes (numeric traits); ^d^ results for the nominal phenotypes (nominal traits); ^e^ Effective Number of Different Populations (*ENDP*) calculated with the assignment-based *KB* distances between populations; ^f^ extent of differentiation (*ED*) among populations measured with the normalized Effective Number of Different Populations (*nENDP*).

**Table 3:** Descriptive statistics of normalized singularity measures for *D. dahli* and *D. portschinskii*.

	*D. dahli*/*D. portschinskii* ^a^		
	SMMv ^b^	Numeric ^c^	Nominal ^d^
Mean, *M*	0.052/**0.240**	0.130/**0.208**	0.559/**0.563**
Standard Deviation, *SD*	0.032/**0.040**	0.041/**0.041**	0.049/**0.064**
Relative range, *RR* ^e^	0.970/**0.446**	0.716/**0.525**	0.389/**0.366**
Coefficient of variation, *CV* ^f^	0.615/**0.167**	0.315/**0.197**	**0.088/0.114**

^a^ estimates for *D. portschinskii* are shown in **bold**; ^b^ results for the STR genotypes assuming the Stepwise Mutation Model with variable mutation rate (SMMv) of STR evolution; ^c^ results for the numeric phenotypes (numeric traits); ^d^ results for the nominal phenotypes (nominal traits); ^e^ $RR=\left(max-min\right)/max$, where max and min are maximum and minimum singularity estimates for the corresponding type of data; ^f^ CV=SD/M.

**Table 4:** Association between the phenotypic dissimilarities and environmental variables on the locality and individual levels.

Species	Phenotypes	Predictors	loc ^d^	R ^e^	*p* ^f^	ind ^g^	R	*p*
	numeric	geodistance ^a^	65	0.152	**0.020**	186	0.088	**0.010**
*D. dahli*	numeric	temperature ^b^	65	0.040	0.231	186	−0.002	0.499
	numeric	rainfall ^c^	65	0.220	**0.021**	186	0.105	**0.026**
	nominal	geodistance	65	0.071	0.058	186	0.079	**0.000**
*D. dahli*	nominal	temperature	65	−0.047	0.845	186	−0.002	0.529
	nominal	rainfall	65	0.025	0.326	186	0.000	0.491
	numeric	geodistance	38	0.096	0.088	54	0.106	**0.018**
*D. portschinskii*	numeric	temperature	38	0.155	**0.042**	54	0.048	0.259
	numeric	rainfall	38	0.100	0.118	54	0.046	0.250
	nominal	geodistance	38	0.096	0.053	54	0.120	**0.002**
*D. portschinskii*	nominal	temperature	38	0.092	0.095	54	0.098	**0.045**
	nominal	rainfall	38	0.113	0.054	54	0.093	0.051

^a^ geographic distance between the localities; ^b^ average summer temperature (BIO10); ^c^ average annual rainfall (BIO12); ^d^ number of studied localities; ^e^ Mantel correlation coefficient; ^f^ significance *p*-value; p≤0.05 are shown in bold; ^g^ number of analyzed individuals.

**Table 5:** Locality-level dbRDA tests of phenotypic dissimilarity explained by climatic variables after dbMEM spatial screening.

Species	Phenotype	R^2^		*p*-Value		
		Model ^a^	Adjusted ^b^	Global ^c^	Temperature ^d^	Rainfall ^e^
*D. dahli*	numeric	0.051	0.015	0.005	0.098	0.015
	nominal	0.041	0.005	0.122	0.618	0.023
*D. portschinskii*	numeric	0.185	0.125	0.001	0.035	0.002
	nominal	0.094	0.027	0.101	0.363	0.069

^a^ proportion of variation in the phenotypic dissimilarity matrix; ^b^ R^2^ corrected for the number of predictors and sample size; ^c^ permutation-based significance of the full dbRDA model; ^d,e^ permutation-based term-level probabilities for the corresponding climatic predictors: ^d^—average summer temperature (BIO10); ^e^—average annual rainfall (BIO12).

## Data Availability

All data generated or analyzed during this study are included in this article (and its Supplementary Tables).

## Supplementary Materials

The following supporting information can be downloaded at: https://www.mdpi.com/article/10.3390/biology15110882/s1, Table S1. Original data for 186 specimens of *Darevskia dahli* and 54 *D. portschinskii*: DD—*D. dahli*, DP—*D. portschinskii*. Sample—individual code of the studied specimens. Numeric variables (normalized): SC-CT—the number of scales around the central temporal scale; ULb—the number of the upper labials; CT-UL6—the number of the scales between the central temporal and sixth upper; TM-UT—the number of the scales between the tympanal and upper temporal; TM-UL7—the number of the scales between the tympanal and seventh upper labial; CT-PPO3—the number of the scales between the central temporal and the third scale of the second road of postorbitals; CT-UT—the number of the scales between the central temporal and the closest upper temporal; SC-AN—the number of the scales in contact with the anal scale; FP—the number of the femoral pores on the left leg. Nominal variables: CT-SHAPE—the shape of the central temporal scale (round or angular); CT-ORIENT—the orientation of the CT: round, or oval—horizontally or vertically elongated; TM-SHAPE—the structure of the tympanal scale (single or divided); PCT—post-CT (single or divided); PO2-PPO3—postorbital and post-postorbital (not in contact, in contact, or overlapping); (f) UL3—upper labial 3 (single or divided); CPA-SHAPE—shape of the CPA—absent, small, large, broad; PRF—prefrontal (side smooth, side angled, scale between the prefrontals); IP—interparietal (parallel or angled lines, concaved, broken lines); IPT—interparietal thickness (IPT). BIO10—average temperature of the warmest quarter at a locality; BIO12—annual rainfall level at a locality. Table S2. The spectrum of normalized singularity estimates for both studied species and six metapopulations.
